# Population screening for hereditary and familial cancer syndromes in Valka district of Latvia

**DOI:** 10.1186/1897-4287-8-8

**Published:** 2010-10-29

**Authors:** Andrejs Vanags, Ilze Štrumfa, Andris Gardovskis, Viktors Borošenko, Arnis Āboliņš, Uldis Teibe, Genadijs Trofimovičs, Edvīns Miklaševičs, Jānis Gardovskis

**Affiliations:** 1 Hereditary Cancer Institute, Rīga Stradiņš University, Dzirciema Street 16, LV 1007, Riga, Latvia; 2 Department of Physics, Rīga Stradiņš University, Dzirciema Street 16, LV 1007, Riga, Latvia

## Abstract

**Background:**

The growing possibilities of cancer prevention and treatment as well as the increasing knowledge about hereditary cancers require proper identification of the persons at risk. The aim of this study was to test the outcome of population screening in the scientific and practical evaluation of hereditary cancer.

**Methods:**

Population screening for hereditary cancer was carried out retrospectively in a geographic area of Latvia. Family cancer histories were collected from 18642 adults representing 76.6% of the population of this area. Hereditary cancer syndromes were diagnosed clinically. Molecular testing for *BRCA1 *founder mutations 300 T/G, 4153delA and 5382insC was conducted in 588 persons who reported at least one case of breast or ovary cancer among blood relatives.

**Results:**

Clinically, 74 (0.40%; 95% confidence interval (CI): 0.32 - 0.50%) high-risk and 548 (2.94%, 95% CI: 2.71 - 3.19) moderate-risk hereditary cancer syndromes were detected covering wide cancer spectrum. All syndromes were characterised by high cancer frequency among blood relatives ranging 8.6 - 46.2% in contrast to spouse correlation of 2.5 - 3.6%. The mean age of cancer onset ranged 38.0 - 72.0 years in different syndromes. The *BRCA1 *gene mutations were identified in 10 (1.7%; 95% CI: 0.9 - 3.1%) probands. Families with established BRCA1 gene founder mutations were identified with the frequency 1:2663 clinically screened persons.

**Conclusions:**

Population screening is a useful practical tool for the identification of persons belonging to families with high frequency of malignant tumours. The whole hereditary and familial cancer spectrum along with the age structure was identified adjusting follow-up guidelines. Another benefit of the population screening is the possibility to identify oncologically healthy persons belonging to hereditary and familial cancer families so that appropriate surveillance can be offered. Clinical diagnostics is appropriate for population screening purposes; molecular investigation provides additional information. In collaboration with family doctors, the screening is technically manageable as characterised by high compliance.

## Background

Development in cancer research has brought not only expanding knowledge of cancer biology but also improved treatment results. Even thought the number of cancer deaths has decreased by 9% in the European Union (EU) between 1985 and 2000, oncological diseases remain an important cause of mortality and morbidity [1]. The number of death due to cancer was estimated 1.12 million in the EU in 2000 [1]. It has been proven that hereditary background can be found for practically all cancers [2]. In 5-10% of common cancers, this hereditary basis represents an inherited single gene mutation with high penetrance [3]. The cancer risk for a healthy person can rise significantly in the presence of a pathogenic mutation with high penetrance [4]. Furthermore, hereditary cancers arise early in life [4,5] and naturally affect several blood relatives. Thus, hereditary cancer determines part of the excessive risk conferred by positive family history of concordant cancer [6-10] making the family history a predictive factor. In order to prevent this group of tumours, people at increased hereditary cancer risk need to be identified earlier. However, this requires a well-planned strategy and the essential tools for detection of people at high risk.

The scientific methods of identifying the hereditary basis of a cancer include analysis of family cancer history as well as molecular tests in order to reveal pathogenic high-penetrance mutations [4,5]. The cancer family history practically can reveal to identify cancer family syndromes characterized by monogenic dominant inheritance pattern with high penetrance. It can be subjected to various bias like denial of serious problems, lack of sufficient knowledge, poor compliance, inability to express the information correctly and others. The suggestion to check the family history data provided by the patient is reasonable [11]. However, high accuracy of reporting cancers, reaching 83% for first-degree relatives, has been described [12,13]. The molecular genetic testing provides objective data but can be impeded by sensitivity restrictions and presence of new or unknown mutation. Thus, absolute efficiency of any screening program cannot be expected.

The best known approach in order to identify hereditary cancer is careful analysis of the family cancer history and search for mutations in a patient receiving tumour treatment [5]. Thus, the healthy family members gain the possibility to estimate their own cancer risk and undergo appropriate diagnostic procedures. Within the present article, this approach is designated in short as hospital screening. Population-based hospital screening provides an insight into the importance of the problem in the local population. An alternative way to detect hereditary cancer is individual testing of persons inquiring about their own cancer risk. This may lead to early diagnostics and/or psychological support.

The third alternative for the diagnostics of hereditary cancer is the population screening - the strategy that targets the whole adult population within a region in order to find out families with increased cancer risk. Hypothetically, the benefits of such approach include revealing of persons at risk before the tumour development. Also, all possible hereditary cancer syndromes can be diagnosed independently of the predominant location. However, population screening demands time, experienced staff and financial resources. Results on population screening for hereditary cancer are very limited [14].

The aim of this study was to test the population screening approach and its success in detecting hereditary cancer in the northeast of Latvia.

## Methods

**Figure 1:**
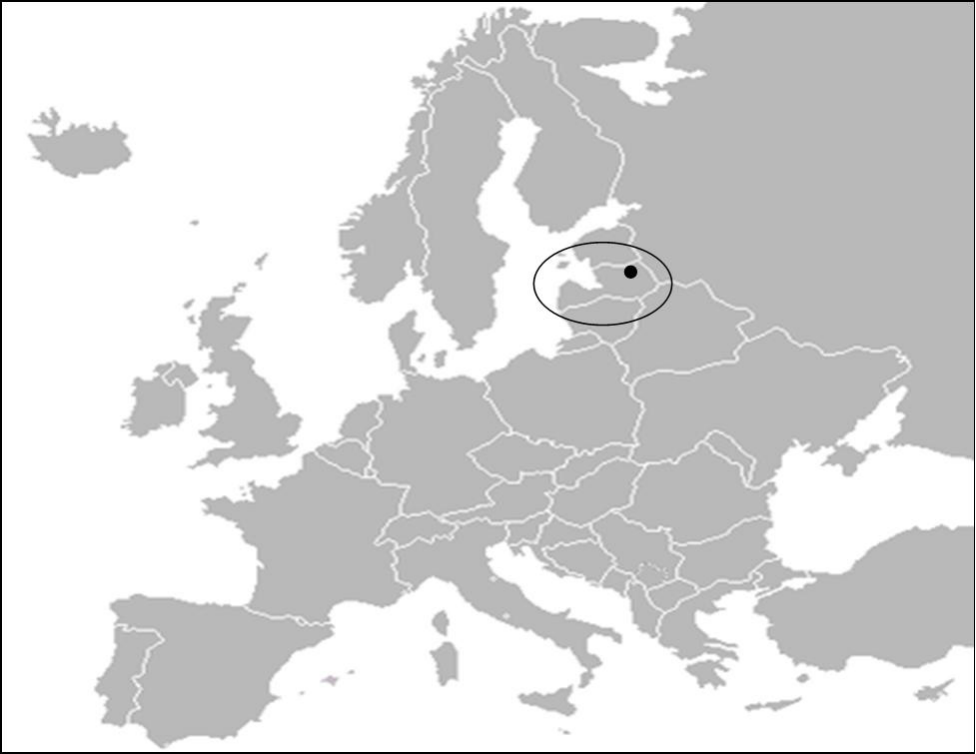
Geographic localisation of Latvia (encircled) with Valka district (dot).

The investigation was designed as population screening for hereditary cancer in the Valka district - a geographical area in the northeast of Latvia (Figure 1). Family cancer histories were collected from 18642 adult inhabitants of Valka district representing 76.6% of the adult population. The study was carried out in close collaboration with family physicians from September 2005 until June 2007. All people were enrolled in the study regardless of gender, ethnicity or health status.

Information on family cancer history was collected using a questionnaire that was previously tested in hospital screening [15]. The participants were asked if his/her blood relatives have had any tumour. In case of an affirmative answer, the participants were asked for the localisation of the tumour and the age of patient at the time of tumour diagnosis. If the patient has died because of the tumour the age at death was recorded as well. In order to verify the presence and location of malignant tumour, information was retrieved (if available) regarding the treatment modalities (e.g. radiation therapy and chemotherapy, extent of operation). The interview took an estimated 45 minutes to complete.

The filled-in questionnaires were analysed by physicians with experience in hereditary cancer diagnostics. The applied diagnostic criteria of the hereditary and familial cancer syndromes are presented in Table 1. Within the frames of the present study, cases corresponding to strict criteria [15,16] were classified into group 1 (g1), but cases diagnosed by more relaxed criteria - into group 2 (g2). The combined group was designated g3.

**Table 1:** Diagnostic criteria used in the population screening for hereditary cancer.

Hereditary syndrome	Diagnostic criteria
Hereditary non-polyposis colorectal cancer, group 1^a ^(HNPCC/g1)	Amsterdam II criteria [15]
HNPCC, group 2 (HNPCC/g2)	At least 2 first degree relatives with HNPCC associated cancer (colorectal, endometrial, small bowel, ureteric, renal pelvis) and at least one cancer diagnosed before age 50
Familial colorectal cancer, group 2 (FCC/g2)	Colorectal cancer in at least two first or second degree relatives. HNPCC and familial adenomatous polyposis should be excluded
Hereditary breast cancer, group 1 (HBC/g1)	At least 3 breast cancer patients in family and one of those patients is first degree relative to other two or second degree relative through male
Hereditary breast cancer, variety 1, group 2 (HBC1/g2)	At least one of the following criteria: 1) Breast cancer diagnosed under the age of 40; 2) Medullary or atypical medullary breast cancer; 3) Male breast cancer; 4) Bilateral breast cancer, one of them diagnosed under the age of 50.
Hereditary breast cancer, variety 2, group 2 (HBC2/g2)	Two breast cancer cases among first degree relatives (or second degree through male)
Hereditary ovarian cancer, group 1 (HOC/g1)	At least 3 ovarian cancer cases in family and one of those patients is first degree relative to other two or second degree relative through male
Hereditary ovarian cancer, group 2 (HOC/g2)	Two ovarian cancer cases among first degree relatives
Hereditary breast-ovarian cancer, group 1 (HBOC/g1)	At least 3 breast or ovarian cancer patients in family at any age and one of those patients is first degree relative to other two or second degree relative through male
Hereditary breast-ovarian cancer, variety 1, group 2 (HBOC1/g2)	Breast and ovarian cancer in the same individual at any age
Hereditary breast/ovarian cancer, variety 2, group 2 (HBOC2/g2)	One breast cancer diagnosed under the age of 50 and one ovarian cancer diagnosed at any age among first degree relatives or second degree relatives through male
Hereditary endometrial cancer, group 1 (HEC/g1)	At least 3 first degree relatives with endometrial cancer and at least one of them diagnosed before age of 50
Hereditary endometrial cancer, group 2 (HEC/g2)	Two first degree relatives with endometrial cancer and at least one of them diagnosed before age of 50
Familial endometrial cancer, group 1 (FEC/g1)	At least 3 first degree relatives with endometrial cancer. HEC/g1 should be excluded
Familial endometrial cancer, variety 1, group 2 (FEC1/g2)	At least 2 first degree relatives with endometrial cancer. HEC/g2 should be excluded
Familial endometrial cancer, variety 2, group 2 (FEC2/g2)	At least 2 second degree relatives with endometrial cancer
Familial lung cancer, group 1 (FLC/g1)	At least 3 first degree relatives with lung cancer
Familial lung cancer, group 2 (FLC/g2)	Two first degree relatives with lung cancer
Hereditary gastric cancer, group 1 (HGC/g1)	At least 3 first degree relatives with gastric cancer
Hereditary gastric cancer, group 2 (HGC/g2)	Two first degree relatives with gastric cancer
Hereditary prostate cancer, group 1 (HPC/g1)	At least 3 blood relatives with prostate cancer at any age or 2 blood relatives with prostate cancer diagnosed before age of 55 in both of them
Hereditary prostate cancer, group 2 (HPC/g2)	Two blood relatives with prostate cancer at any age or a case of prostate cancer diagnosed before age of 55
Familial brain tumour, group 1 (FBT/g1)	At least 3 first degree relatives with brain tumour
Familial brain tumour, group 2 (FBT/g2)	Two first degree relatives with brain tumour
Familial malignant haematological tumour, group 1 (FHemT/g1)	At least 3 first degree relatives with malignant haematological tumour
Familial malignant haematological tumour, group 2 (FHemT/g2)	Two first degree relatives with malignant haematological tumour
Familial pancreatic tumour, group 1 (FPan/g1)	At least 2 first degree relatives with pancreatic tumour or melanoma
Familial urinary bladder cancer, group 1 (FBlaC/g1)	At least 3 first degree relatives with urinary bladder cancer
Familial urinary bladder cancer, group 2 (FBlaC/g2)	Two first degree relatives with urinary bladder cancer
Other familial cancer syndromes	At least 3 first degree relatives with concordant cancer

^a^groups 1 and 2 were defined for the use solely within the presented study

Patients diagnosed with any hereditary cancer syndrome were invited for additional consultation in order to confirm the diagnosis and to discuss the possibilities of molecular diagnostics. Mutually related families were identified in order to prevent repeated inclusion of any affected person in further calculations.

The study was approved by the Central Commission of Medical Ethics of Latvia. Written informed consent was obtained from all patients.

The statistic evaluation was carried out by CIA software [17]. The population prevalence of any hereditary cancer syndrome was calculated as the ratio between the number of clinically diagnosed individuals and the whole group. The frequency of cancer among blood relatives was estimated. Size of family was characterised by number of blood relatives in the affected genetic line. The course of malignant tumour was characterised by the data about the age of tumour diagnostics and age of tumour-related death. Using the data about the total number of cancers reported in the kindred by all recruited persons and the number of cancers in the families affected by hereditary cancer syndrome, the hereditary cancer burden was calculated for each location.

Molecular examination for the *BRCA1 *founder mutations 300T/G, 4153delA and 5382insC as described by Gorski et al. [18] was offered to the participants of the study reporting at least one case of breast or ovarian cancer in their family history. The molecular examination was provided for 588 patients.

## Results

**Figure 2:**
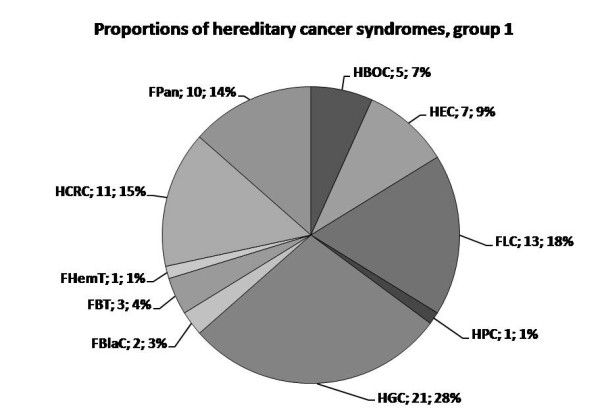
**The reciprocal proportions of hereditary cancer syndromes within group 1**. Abbreviations of the syndromes are followed by absolute number of identified probands with the corresponding diagnosis and the proportion within the group 1. HCRC is used to comprise both HNPCC and FCC.

**Figure 3:**
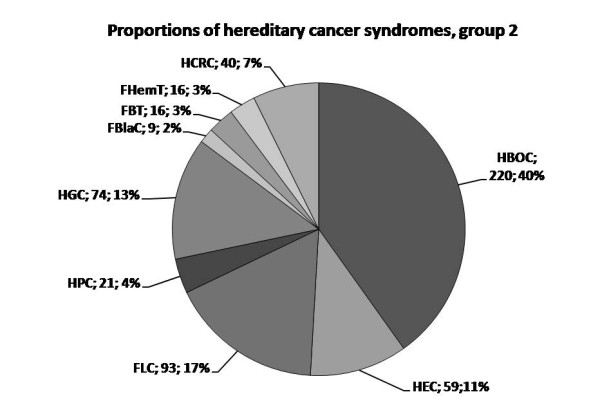
**The reciprocal proportions of hereditary cancer syndromes within group 2**. Abbreviations of the syndromes are followed by absolute number of identified probands with the corresponding diagnosis and the proportion within the group 2. HCRC is used to comprise both HNPCC and FCC.

The population screening by clinical criteria identified 74 (0.40%; 95% confidence interval (CI): 0.32 - 0.50%) hereditary cancer cases of group 1. Hereditary gastric cancer (HGC) was the most common syndrome, followed by familial lung cancer (FLC) and hereditary non-polyposis colorectal cancer (HNPCC) syndromes (Figure 2). In addition, 548 (2.94%, 95% CI: 2.71 - 3.19%) probands reported family history corresponding to group 2 criteria (Table 2). Here, breast and ovarian cancers were dominating locations, followed by lung cancer (Figure 3). Renal and testicular cancers were among the locations not reported by the applied criteria. The population prevalence of each hereditary and familial cancer syndrome ranged 0.005 - 0.11% in group 1 and 0.05 - 1.18% in group 2 (Table 2).

**Table 2:** Characteristics of hereditary cancer syndromes by population prevalence, proband's health status and frequency of index cancers among blood relatives.

Diagnosis	Population prevalence		Affected probands		Cancer frequency	
	
	Value,%	95% CI,%	Fr,%	95% CI,%	Value,%	95% CI,%
HNPCC/g1	0.06	0.03 - 0.10	18.2	5.1 - 47.7	30.1	23.3 - 38.0
					15.8^CRC^	10.7 - 22.5^CRC^
					22.4^EF^	14.8 - 32.3^EF^
HNPCC/g2	0.11	0.07 - 0.17	5	0.9 - 23.6	15.5	11.6 - 20.3
					10.6^CRC^	7.4 - 14.8^CRC^
					9.6^EF^	5.7 - 15.8^EF^
HNPCC/g3	0.17	0.12 - 0.24	9.7	3.3 - 24.9	19.3	16.5 - 22.5
					12.4^CRC^	9.6 - 15.9^CRC^
					14.5^EF^	10.5 - 19.8^EF^
FCC/g2	0.11	0.07 - 0.17	5	0.9 - 23.6	17.0	12.8 - 22.3
HB+OC/g1	0.03	0.01 - 0.06	20	3.6 - 62.4	24.6 ^F^	15.5 - 36.7 ^F^
HBC1/g2	0.63	0.52 - 0.75	4.3	1.8 - 9.6	8.6	7.2 - 10.2
					16.3^F^	13.8 - 19.1^F^
HBC2/g2	0.34	0.27 - 0.44	18.8	11.1 - 30.0	31.8^F^	27.5 - 36.4^F^
HBOC1/g2	0.03	0.02 - 0.07	0	0 - 39.0	19.3^F^	9.2 - 36.3 ^F^
HBOC2/g2	0.16	0.11 - 0.22	20.7	9.8 - 38.4	30.8^F^	25.0 - 37.3^F^
HOC/g2	0.008	0.001 - 0.06	0	0 - 49.0	36.4^F^	19.7 - 57.0^F^
HB+OC/g2	1.18	1.04 - 1.35	10.5	7.1 - 15.2	12.6 ^F^	11.4 - 13.9 ^F^
					23.6^F^	21.4 - 25.9^F^
HEC/g1	0.03	0.01 - 0.06	0	0 - 43.3	41.5^F^	27.8 - 56.6^F^
HEC/g2	0.14	0.10 - 0.20	15.4	6.2 - 33.5	32.2^F^	25.7 - 39.4 ^F^
FEC/g1	0.01	0.003 - 0.04	0	0 - 65.8	46.2 ^F^	23.2 - 70.9 ^F^
FEC1/g2	0.13	0.09 - 0.19	12.5	4.3 - 31.0	28.7 ^F^	22.4 - 36.0 ^F^
FEC2/g2	0.05	0.03 - 0.09	11.1	2.0 - 43.5	32.4 ^F^	22.4 - 44.2 ^F^
H+FEC/g1	0.04	0.02 - 0.08	0	0 - 35.4	42.6 ^F^	36.3 - 55.8 ^F^
H+FEC/g2	0.32	0.25 - 0.41	13.6	7.0 - 24.5	30.8 ^F^	26.5 - 35.4 ^F^
H+FEC g3	0.35	0.28 - 0.45	14.3	7.4 - 25.7	41.0 ^F^	36.1 - 46.2 ^F^
HGC/g1	0.11	0.07 - 0.17	4.8	0.8 - 22.7	25.2	20.6 - 30.4
HGC/g2	0.40	0.32 - 0.50	0	0 - 4.9	16.0	13.8 - 18.5
HGC/g3	0.51	0.42 - 0.62	1.1	0.2 - 5.7	18.2	16.2 - 20.5
FLC/g1	0.07	0.04 - 0.12	0	0 - 22.8	25.5	19.3 - 32.8
FLC/g2	0.50	0.41 - 0.61	0	0 - 4.0	17.2	15.0 - 19.7
FLC/g3	0.57	0.47 - 0.69	0	0 - 3.5	18.3	16.2 - 20.7
HPC/g1	0.005	0.001 - 0.03	100	20.7 - 100	21.4^M^	7.6 - 47.6^M^
HPC/g2	0.11	0.07 - 0.17	19.0	7.7 - 40.0	22.2^M^	16.4 - 29.4^M^
HPC/g3	0.12	0.08 - 0.18	22.7	10.1 - 43.4	22.2	16.5 - 29.0
FPanC/g1	0.05	0.03 - 0.10	10	1.8 - 40.4	14.7	9.1 - 22.9
FBlaC/g1	0.01	0.003 - 0.04	0	0 - 65.8	31.6	15.4 - 54.0
FBlaC/g2	0.05	0.03 - 0.09	0	0 - 29.9	20.0	11.8 - 31.8
FBlaC/g3	0.06	0.03 - 0.11	0	0 - 25.9	22.8	14.9 - 33.2
FHemT/g1	0.005	0.001 - 0.03	0	0 - 79.3	30.8	12.7 - 57.6
FHemT/g2	0.09	0.05 - 0.14	6.3	1.1 - 28.3	15.4	11.2 - 20.9
FHemT/g3	0.09	0.06 - 0.15	5.9	1.0 - 27.0	16.3	12.1 - 21.2
FBT/g1	0.02	0.005 - 0.05	0	0 - 56.2	32.3	18.6 - 49.9
FBT/g2	0.09	0.05 - 0.14	0	0 - 19.4	14.4	10.4 - 19.5
FBT/g3	0.10	0.07 - 0.16	0	0 - 16.8	16.5	12.5 - 21.5

^CRC ^colorectal cancer; ^EF ^endometrial cancer in female; ^F ^in female; ^M ^in male

CI, confidence interval; Fr, frequency; HB+OC, combined group of hereditary breast and/or ovarian cancer; H+FEC, combined group of hereditary and familial endometrial cancer

The identified pedigrees of hereditary and familial cancer syndromes were characterised by generally high frequency of malignant tumours among the blood relatives as shown in Table 2. The highest cancer frequency was recorded in the following groups: HEC/g1 (41.5%, 95% CI: 27.8 - 56.6%), the whole hereditary and familial endometrial cancer group (41.0%, 95% CI: 36.1 - 46.2%), HNPPC/g1 (30.1%, 95% CI: 23.3 - 38.0%), FLC/g1 (25.5%, 95% CI: 19.3 - 32.8%) and HGC/g1 (25.2%, 95% CI: 20.6 - 30.4%). In contrast, the spouse correlation was as low as 2.5% (95% CI: 0.7 - 8.6%) in FLC/g3 families and 3.6% (95% CI: 1.2 - 10.1%) in HGC/g3 families.

Comparison of cancer frequency between groups 1 and 2 was carried out for each location. For some locations statistically significant difference was found confirming the patient stratification. Thus, in case of HNPCC and HGC the cancer frequency was 30.1% (95% CI: 23.3 - 38.0%) vs. 15.5% (95% CI: 11.6 - 20.3%) and 25.2% (95% CI: 20.6 - 30.4%) vs. 16.0% (95% CI: 13.8 - 18.5%), respectively. The importance of both g1 and g2 proper identification was demonstrated by following data. Close frequencies of colorectal cancer was observed in HNPCC/g1, HNPCC/g2 and FCC/g2, namely 15.8% (95% CI: 10.7 - 22.5%), 10.6% (95% CI: 7.4 - 14.8%) and 17.0% (95% CI: 12.8 - 22.3%). Similarly, individual syndromes of hereditary or familial endometrial cancer showed close frequencies of endometrial cancer although significant difference was observed among combined g1 and g2 endometrial cancer syndromes (Table 2).

The health status of the probands was analysed as one of the major aspects characterising both the population screening approach, and particular hereditary cancer syndromes (Table 2). Low frequency of the affected probands was recorded in FLC (0%, 95% CI: 0 - 3.5%), in FBT (0%, 95% CI: 0 - 16.8%), in HGC (1.1%, 95% CI: 0.2 - 5.7%) as well in FBlaC (0%, 95% CI: 0 - 25.9%). The highest frequency of affected probands was observed in HPC (22.2%, 95% CI: 16.5 - 29.0%) and in the whole group of hereditary and familial endometrial cancer (14.3%, 95% CI: 7.4 - 25.7%).

There was statistically significant difference between the size of family diagnosed with a group 1 or 2 hereditary cancer syndrome and families with non-diagnostic findings (Table 3).

**Table 3:** The reported family size in hereditary or familial cancer syndromes and other status of family cancer history.

Group	The number of blood relatives			
	
	Interval	Mean	SD	95% CIM
g1	7 - 29	13.6	4.9	12.2 - 15.0
g2	3 - 47	12.2	4.8	11.7 - 12.7
Not diagnostic	4 - 25	9.5	3.8	8.9 - 10.1

SD, standard deviation; CIM, confidence interval for the mean.

The mean age of cancer diagnostics and cancer-related death is presented in Table 4. The lowest mean age of cancer diagnostics was found in HBC1/g2 (38.0 years, 95% CI: 36.2 - 39.7 years), at least partially due to the selection criteria. The mean age was low in FBT (43.9 years, 95% CI: 35.0 - 52.8 years) and in FHemT (47.5 years, 95% CI: 38.9 - 56.1 years), at least partially due to the cancer occurrence in childhood. The mean age was below 50 years in few other groups: HBC/g1 (47.5 years, 95% CI: 37.1 - 57.8 years), in HEC/g2 (48.5 years, 95% CI: 44.4 - 52.6 years), in HBOC1/g2 (48.8 years, 95% CI: 44.2 - 53.3 years), in HOC/g1 (49.7 years, 95% CI: 4.0 - 95.4 years) and in HNPCC/g1-related endometrial cancer (48.4 years, 95% CI: 43.4 - 53.4 years). FBlaC/g3 was characterised by high mean age of tumour diagnostics (70.7 years, 95% CI: 66.7 - 74.7 years, respectively) as well as HPC/g1 (72.0 years, 95% CI: 67.0 - 76.9 years) and FCC/g2 (72.0 years, 95% CI: 67.3 - 76.7 years). The proportion of cases occurring before the age of 50 was statistically significantly higher in the hereditary group than in whole population for colorectal, endometrial, breast, lung cancer and haematological tumours (Table 5).

**Table 4:** The age of hereditary cancer diagnostics and cancer-related death by population screening data.

Syndrome	Age of diagnosis		Age of death	
	
	Interval (years)	Mean (95% CIM)	Interval (years)	Mean (95% CIM)
HBC/g1	40 - 55	47.5 (37.1 - 57.8)	50 - 60	54.7 (50.0 - 59.4)
HBC1/g2	20 - 70	38.0 (36.2 - 39.7)	26 - 78	44.7 (41.7 - 47.7)
HBC2/g2	25 - 82	51.8 (48.9 - 54.6)	25 - 66	60.9 (56.7 - 65.1)
HBOC/g1	34 - 82	61.0 (46.9 - 75.0)	58 - 85	71.4 (54.9 - 87.9)
HBOC1/g2	40 - 60	48.8 (44.2 - 53.3)	47 - 69	54.3 (42.3 - 66.3)
HBOC2/g2	18 - 86	56.6 (51.8 - 61.4)	23 - 87	66.1 (61.2 - 71.0)
HOC/g1	34 - 70	49.7 (4.0 - 95.4)	72	72
HOC/g2	45 - 70	54.2 (46.4 - 61.9)	47 - 72	57.2 (50.1 - 64.3)
HNPCC/g1	30 - 77	54.2 (50.2 - 58.2)	28 - 89	61.7 (54.2 - 69.2)
CRC	36 - 77	59.3 (53.8 - 64.8)	28 - 89	61.5 (52.9 - 70.0)
E	30 - 65	48.4 (43.4 - 53.4)	37 - 72	NA
HNPCC/g2	27 - 82	53.7 (49.1 - 58.3)	28 - 88	55.5 (49.5 - 61.5)
CRC	28 - 82	55.2 (49.1 - 61.3)	32 - 88	56.7 (49.9 - 63.5)
E	27 - 72	50.5 (43.0 - 58.0)	28 - 73	51.2 (33.1 - 69.3)
FCC/g2	41 - 89	72.0 (67.3 - 76.7)	52 - 90	76.3 (73.1 - 79.5)
HEC/g1	40 - 75	52.1 (47.2 - 57.0)	44 - 76	57.7 (49.6 - 65.8)
HEC/g2	30 - 81	48.5 (44.4 - 52.6)	35 - 87	58.7 (53.6 - 63.8)
FEC/g1	52 - 90	66.3 (63.0 - 69.6)	54 - 91	71.7 (68.6 - 74.8)
FEC1/g2	60 - 78	67.0 (53.1 - 80.9)	65 - 86	79.2 (69.0 - 84.4)
FEC2/g2	26 - 82	57.6 (49.9 - 65.3)	26 - 83	63.3 (54.7 - 71.9)
FLC/g1	35 - 78	56.0 (53.0 - 59.0)	36 - 79	57.1 (54.1 - 60.1)
FLC/g2	18 - 90	58.5 (56.1 - 60.9)	13 - 90	61.2 (59.1 - 63.3)
FLC/g3	18 - 90	57.9 (55.9 - 59.9)	13 - 90	61.2 (58.5 - 62.1)
HGC/g1	30 - 83	56.9 (53.4 - 66.3)	30 - 90	58.3 (55.3 - 61.3)
HGC/g2	34 - 95	62.5 (60.1 - 64.8)	37 - 96	65.6 (63.4 - 67.6)
HPC/g1	70 - 74	72.0 (67.0 - 76.9)	72	NA
HPC/g2	35 - 75	56.8 (52.8 - 60.8)	37 - 80	59.7 (54.6 - 64.8)
HPC/g3	35 - 75	57.7 (53.3 - 62.1)	37 - 80	60.7 (55.0 - 66.4)
FBlaC/g1	60 - 75	67.5 (53.7 - 81.3)	65 - 75	70.3 (65.6 - 75.0)
FBlaC/g2	61 - 87	71.8 (67.3 - 76.3)	72 - 92	79.3 (74.1 - 84.5)
FBlaC/g3	60 - 87	70.7 (66.7 - 74.7)	65 - 92	75.7 (71.6 - 79.8)
FHemT/g1	45 - 61	50.7 (28.4 - 73.0)	46 - 50	48.0 (22.6 - 73.4)
FHemT/g2	3 - 88	47.1 (37.5 - 56.7)	4 - 90	49.9 (39.7 - 60.1)
FHemT/g3	3 - 88	47.5 (38.9 - 56.1)	4 - 86	49.8 (40.5 - 59.1)
FPanC/g1	51 - 72	61.6 (57.3 - 65.9)	51 - 83	63.4 (58.2 - 68.6)
FBT/g1	59 - 60	59.7 (58.3 - 61.1)	50 - 65	59.7 (54.3 - 65.1)
FBT/g2	2 - 77	41.8 (32.0 - 51.6)	2 - 75	45.2 (35.9 - 54.5)
FBT/g3	2 - 77	43.9 (35.0 - 52.8)	2 - 77	47.8 (39.7 - 55.9)

CIM, confidence interval for the mean; CRC, colorectal cancer; E, endometrial cancer; NA, not applicable.

**Table 5:** The proportion of early-onset cancer cases by location in the hereditary group in comparison with the whole studied Valka population.

Location	Hereditary cancers		Valka population		
		
	N	Prop. (95% CI)	N	Prop. (95% CI)	p < 0.05^1^
Colorectal	19/92	20.6 (13.6-30.0)	43/777	5.8 (4.3-7.7)	Yes
Endometrial	51/181	28.2 (22.1-35.1)	158/1094	15.1(13.1-17.5)	Yes
Breast	170/310	54.8 (49.3-60.3)	279/1364	21.8(19.7-24.2)	Yes
Ovarian	16/51	31.4 (20.3-45.0)	39/156	27.1 (20.5-34.9)	No
Lung	35/232	15.0 (11.1-20.3)	147/1795	8.3 (7.1-9.6)	Yes
Stomach	20/225	8.9 (5.8-13.3)	162/1615	10.2 (8.8-11.8)	No
Pancreas	0/21	0 (0-15.5)	19/302	6.3 (4.1-9.6)	No
Prostate	4/37	10.8 (4.3-24.7)	12/499	2.6 (1.5-4.4)	No
Haematologic	13/37	35.1 (21.8-51.2)	115/687	16.9 (14.3-19.9)	Yes
Urinary bladder	0/24	0 (0-13.8)	11/249	4.6 (2.6-8.1)	No
Brain	10/43	23.3 (13.1-37.3)	14/96	14.9 (9.1-23.5)	No

^1^Difference statistically significant at p < 0.05

N, number of early-onset cases/whole number of reported cases; Prop., proportion.

The hereditary cancer burden by location is shown in Table 6. All the most frequent cancer locations are characterized by hereditary cancer burden exceeding 10% at group 3 level. The hereditary cancer burden averaged 2.4 - 4.7% for frequently encountered tumours as colorectal, endometrial, ovarian, lung and gastric cancer in group 1.

**Table 6:** Hereditary cancer burden by location.

Origin of the tumour	Hereditary cases, group 3		Hereditary cases, group 1	
	
	Fraction,%	95% CI,%	Fraction,%	95% CI,%
Colorectal	11.8	9.8 - 14.3	3.0	2.0 - 4.4
Endometrial	16.5	14.5 - 18.9	3.8	2.9 - 5.1
Breast	25.0	22.7 - 27.4	0.8	0.4 - 1.4
Ovarian	35.4	28.1 - 43.5	3.5	1.5 - 7.9
Lung	12.9	11.5 - 14.6	2.4	1.8 - 3.2
Stomach	13.8	12.2 - 15.6	4.7	3.8 - 5.9
Pancreas	6.3	4.1 - 9.6	6.3	4.1 - 9.6
Melanoma	20.0	8.1 - 41.6	10.0	2.8 - 30.1
Prostate	7.4	5.4 - 10.1	0.6	0.2 - 1.8
Haematologic	5.4	3.9 - 7.3	0.6	0.2 - 1.5
Urinary bladder	9.6	6.6 - 13.9	2.4	1.1 - 5.2

During the population screening in Valka district 10 *BRCA1 *gene mutations in 7 families were found, representing 1.7% (95% CI: 0.9 - 3.1%) of the molecularly screened group, and 0.05% (95% CI: 0.03 - 0.1%) of the screened Valka population. None of the mutation carriers was diagnosed by clinical criteria of group 1 (Table 7).

**Table 7:** Relation between the presence of *BRCA1 *mutation and clinical data.

Hereditary cancer syndromes	Tested persons	*BRCA1 *mutation
Hereditary breast - ovarian cancer, group 1	5	0 0% (95% CI: 0 - 43.5%)
Hereditary breast - ovarian cancer, group 2	153	2 1.3% (95% CI: 0.4 - 4.6%)
Single case of index cancer in the kindred	430	8 1.9% (95% CI: 0.9 - 3.6%)

The frequency of breast cancer in *BRCA1 *founder mutation carriers was 20%; 95% CI = 5.7 - 51.0] but in tested mutation-negative probands - 10.6% (95% CI = 8.3 - 13.3%). The corresponding incidence rates were 657.9 (95% CI = 79.7 - 2376.5) and 221.8 (95% CI = 169.7 - 284.9) per 100 thousands. There were no ovarian cancer cases in *BRCA1 *mutation carriers, corresponding to the frequency 0% (95% CI = 0 - 27.8%) and incidence rate 0 (95% CI = 0 - 1213) per 100 thousands. In *BRCA1*-mutation negative persons the frequency of ovarian cancer was 1.2% (95% CI = 0.6 - 2.5%) and incidence rate - 25.4 (95% CI = 10.2 - 52.4) per 100 thousands.

## Discussion

For many cancer locations, epidemiologic data suggest that positive family history is a risk factor [6-10]. The identification of the responsible genes and mutations is a major achievement of medical science providing the final evidence of the hereditary nature of a subgroup of cancers as well as practical tool for risk evaluation. However, the molecular analysis is complicated due to genetic heterogeneity, involving unknown mutations and genes, as well as multifactorial inheritance [5]. Technological factors can influence the testing as even sensitive techniques have less than 90% sensitivity for all possible mutations and large deletions can be missed by sequencing [11]. For some tumours, e.g. lung cancer [19], only the susceptibility loci are identified. Despite the lack of comprehensive molecular proof the hypothesis of risk group identification by family history [5] seems reasonable and desirable for cancer eradication.

Against this background the population screening for hereditary cancer is a rather novel approach. It is embarrassed by difficulties including variability of diagnostic criteria [16] and lack of comprehensive molecular genetic testing.

To our best knowledge, only one study on population screening for hereditary cancer [14] is published as yet. In this study carried out in the West Pomeranian region of Poland family cancer history of 1.2 million individuals was evaluated by questionnaire. So results presented in this paper are of great interest. However, further studies with extended statistical analysis are desirable. Expanding the design of population screening, originally provided by Gronwald et al. [14], we have analysed the age of cancer development as well as cancer frequency among blood relatives, health status of probands and hereditary cancer burden by location. The obtained data have both practical significance in cancer prevention and scientific novelty presenting the full spectrum of hereditary cancers.

The performed population screening yielded reasonable number of hereditary and familial cancer syndrome diagnoses. As comprehensive mutation analysis is not possible at the present the high frequency of index cancer among blood relatives in the identified groups can be considered as a risk indicator and also as an evidence of the expedience of the applied criteria. We tested 2 sets of criteria - strict and more relaxed one paralleling the published approach [5]. Both sets identified families with high cancer frequency; therefore we recommend these criteria to identify persons for primary and secondary prevention and surveillance. However, further studies will probably limit the follow-up group as our study had a major drawback: due to historical reasons, older medical documentation was not available necessitating elaborating simple questionnaire in accordance with the reported acceptable rate of accurate reporting cancer in relatives [12].

The clustering of cancer can occur due to chance. The familial risk can also be attributed to hereditary mutations or shared environment and lifestyle factors including heritability of lifestyle. In our study, the spouse correlation in such locations as lung or gastric cancer with significant role of known environmental risk factors [7,20] was lower than the frequency of cancer among blood relatives pointing towards the presence of genetic background.

The majority of the identified probands were healthy people. This approach using simple questionnaire helps to identify healthy persons belonging to families with a high frequency of particular malignant tumours. Surveillance should be advised for most of these people as follow-up is available for several common syndromes, and early diagnostics may have general benefit in reduction of mortality. Furthermore, during population cancer screening in Valka district the mean age of familial cancer onset in different locations was determined facilitating planning of prophylactic health check-up. The crucial value of population screening relies not only on surveillance but also on prevention and adjusted treatment options in the case of established hereditary cancer [21,22]. Prophylactic surgical intervention is possible only after objective verification of the diagnosis [23].

The full spectrum of hereditary cancers was revealed including breast, lung, stomach, large bowel and endometrium as main primary locations. The high proportion of breast, colorectal and gastric cancer among the familial cancers is similar to the findings in Poland [14]. Notably, no kindreds with renal cancer aggregation were identified by the applied criteria. However, these criteria reflect the dominant mode of transmission. A recessive mode of inheritance for renal cancer has been suggested [9] and in this case our criteria based on dominant mode are not applicable.

The full data set of hereditary cancer burden by location in the same group was obtained by population screening. The burden of hereditary colorectal cancer corresponds to the wide published spectrum of 1 - 13% but is lower than average estimates 5 - 6% [24]. As expected, it exceeds the described proportion of families corresponding to Amsterdam criteria [25] although similar proportions have also been reported [26]. The burden of hereditary gastric cancer is higher than estimated by Cisco et al. [27]. Only small fraction of breast cancers corresponds to the strict criteria in contrast to frequent occurrence of relaxed (g2) criteria and hereditary ovarian cancer. As the occurrence of g1 hereditary breast cancer is rare and results from clinical screening and molecular testing do not mutually overlap, the real burden of hereditary breast-ovarian cancer is much higher than identified by g1 criteria. In contrast, the burden of familial lung cancer in our study was 2.4% that is higher than in Japan and USA (0.2 - 0.4%) by the same criteria [19,28]. Notably, the genetically determined predisposition to lung cancer can interact with smoking [19,29] therefore these factors cannot be considered mutually exclusive. The burden of familial pancreatic cancer in our study was 6.3% that is in line with other published results [see for reference [30]].

The population screening for disease-causing mutations [31,32] becomes rational if it corresponds to certain criteria. The disease must be common and serious, with high penetrance and manageable number of predominant mutations. The test must be cheap, acceptable to whole population and an effective surveillance must be feasible [31]. It was shown earlier that *BRCA1 *gene founder mutations 4153delA and 5382insC are common in Latvia [15,33].

In order to fulfil the assumption of the economic efficacy of the screening test, only founder mutations in the *BRCA1 *gene were searched for and testing was offered only to the persons who reported at least one case of breast and/or ovarian cancer in the family. Part of the *BRCA1 *mutations may be missed by this approach. However, in this way we found 10 mutation carriers in 7 families that correspond to 2663 clinically screened persons per one mutation-bearing family. This value is comparable with data obtained by Gronwald et al. [14] who reported 438 mutation-bearing families corresponding to 2873 clinically screened persons per one mutation-bearing family.

The number of *BRCA1 *mutations exceeds the one of probands diagnosed by clinical criteria for hereditary breast-ovarian cancer, group 1. None of the *BRCA1 *mutation carriers was identified by group 1 clinical diagnostic criteria and 8 of them reported only isolated cases of index cancer among blood relatives. Thus, clinical criteria revealed less than half of high-risk persons. The finding of the *BRCA1 *mutations in individuals with no significant family history may be explained by paternal inheritance, lack of knowledge of the family cancer history or small family size. In this study, the inheritance through male was ascertained by criteria. The significance of the family size was demonstrated by higher number of blood relatives in group 1 and 2 in comparison to non-diagnostic family histories.

The *BRCA *mutations are known to be associated with high lifetime breast cancer risk [23]. Although the patient group was already selected for *BRCA1 *testing by positive family history of breast cancer that might include also presence of breast cancer in the individual there is a trend towards higher breast cancer frequency and incidence rate in mutation carriers. The difference does not gain statistical significance due to small sample size and specific group design.

Clinical population screening by relaxed criteria can be highly recommended as it yields high number of persons to whom further surveillance should be advised. This is a practical advantage of population screening for hereditary cancer.

## Conclusions

1. Population screening is a useful practical tool for the identification of persons belonging to families with high frequency of malignant tumours. Another benefit of the population screening is the possibility to identify oncologically healthy persons belonging to hereditary and familial cancer families so that appropriate surveillance can be offered.

2. The population screening based on clinical criteria reveals the full spectrum of hereditary cancers. Hereditary gastric cancer, familial lung cancer, hereditary non-polyposis colorectal cancer and hereditary breast and/or ovarian cancer were among the most common hereditary cancer syndromes.

3. Population screening discloses the age structure of hereditary cancer in the population helping to adjust the surveillance programs.

4. In collaboration with family doctors, the screening is technically manageable as characterised by high compliance (76.6%).

5. Molecular examination is an important tool in the evaluation of individual risk. It should be applied to all persons with at least single breast and/or ovarian cancer case in family if testing for founder mutations is available.

6. Families with established BRCA1 gene founder mutations were identified with the frequency 1:2663 clinically screened persons.

## Competing interests

The authors declare that they have no competing interests.

## Authors' contributions

AV participated in the study design and clinical consultations, performed statistical analysis and wrote the manuscript. IS helped to draft the manuscript. AG and VB participated in clinical consultations. AA participated in the analysis and interpretation of data. UT supervised the statistical analysis. GT helped to draft the manuscript. EM performed the molecular examinations. JG designed and coordinated the study and helped to draft the manuscript. All authors read and approved the final manuscript.
